# Nano Delivery Chitosan-Protein/Hydrolysate of Green Peas Bromelain (PHGPB) Synthesized by Colloidal-Spray Drying Method

**DOI:** 10.3390/polym15112546

**Published:** 2023-05-31

**Authors:** Meilinah Hidayat, Khomaini Hasan, Muhamad Yusuf, Sriwidodo Sriwidodo, Camellia Panatarani, I Made Joni

**Affiliations:** 1 Department of Nutrition, Faculty of Medicine, Universitas Kristen Maranatha, Jalan Suria Sumantri 65, Bandung 40164, West Java, Indonesia; meilinah.hidayat@med.maranatha.edu; 2 Department of Biochemistry, Faculty of Medicine, Universitas Jenderal Achmad Yani, Jalan Terusan Jenderal Sudirman, P.O. Box 148, Cimahi 40531, West Java, Indonesia; k.hasan@lecture.unjani.ac.id; 3 Department of Chemistry, Faculty of Mathematics and Natural Sciences, Universitas Padjadjaran, Jalan Raya Bandung_Sumedang KM 21, Sumedang 45363, West Java, Indonesia; m.yusuf@unpad.ac.id; 4 Department of Pharmaceutics and Pharmaceutical Technology, Faculty of Pharmacy, Universitas Padjadjaran, Jalan Raya Bandung-Sumedang KM 21, Jatinangor 45363, West Java, Indonesia; sriwidodo@unpad.ac.id; 5 Department of Physics, Faculty of Mathematics and Natural Sciences, Universitas Padjadjaran, Jalan Raya Bandung_Sumedang KM 21, Sumedang 45363, West Java, Indonesia; c.panatarani@phys.unpad.ac.id; 6 Functional Nano Powder (FiNder), University Center of Excellence, Universitas Padjadjaran, Jalan Raya Bandung_Sumedang KM 21, Sumedang 45363, West Java, Indonesia

**Keywords:** chronic kidney disease, hydrolysate of green peas bromelain, drugs delivery system

## Abstract

Patients with chronic kidney disease (CKD) suffer persistent decreased kidney function. Previous study of protein hydrolysate of green pea (Pisum sativum) bromelain (PHGPB) has shown promising results as an antifibrotic in glucose-induced renal mesangial culture cells, by decreasing their TGF-β levels. To be effective, protein derived from PHGPB must provide adequate protein intake and reach the target organs. This paper presents a drug delivery system for the formulation of PHGPB using chitosan as polymeric nanoparticles. A PHGPB nano delivery system was synthesized by precipitation with fixed chitosan 0.1 wt.%, followed by a spray drying process at different aerosol flow rates of 1, 3, and 5 L/min. FTIR results showed that the PHGPB was entrapped in the chitosan polymer particles. Homogeneous size and spherical morphology of NDs were obtained for the chitosan-PHGPB with a flow rate of 1 L/min. Our in vivo study showed that the highest entrapment efficiency, solubility, and sustained release were achieved by the delivery system method at 1 L/min. It was concluded that the chitosan-PHGPB delivery system developed in this study improves pharmacokinetics compared to pure PHGPB.

## 1. Introduction

The kidney is a vital organ that removes waste and excess fluid from the body. The kidney also removes acid produced by the body’s cells and maintains a healthy balance of water, salts, and minerals, such as sodium, calcium, phosphorus, and potassium, in the blood. Kidney dysfunction can disrupt fluid and electrolyte balance and cause serious clinical problems. However, it can go undetected until it has progressed to an advanced stage; hence, kidney disease is often considered a silent killer. Common kidney diseases include IgA nephropathy, membranous nephropathy, acute kidney injury, etc. However, kidney disease has gradually become a global health burden. Cases of chronic kidney disease (CKD) continue to increase in Indonesia every year. Data from Riskesdas 2013 shows that the incidence of CKD patients is 0.2 per thousand, but in Riskesdas 2018, it has almost doubled (0.38 per mile) [1]. This is a heavy burden for the Indonesian government, especially in terms of the cost of hemodialysis for CKD patients, which is covered mainly by the Indonesian National Health Insurance Fund (BPJS).

In chronic kidney disease patients, diet and nutrition play an even greater role in managing disease progression. This is because the kidneys are less effective at removing unwanted fluid and managing proper levels of nutrients like calcium, phosphate, and potassium. Therefore, planning a balanced and healthy diet is crucial. The type of protein that does not aggravate kidney damage is a protein in simple or amino acid forms, which is easily absorbed by the body through the enzymatic hydrolysis process and does not produce much waste from protein metabolism to be excreted by the kidneys. Protein Hydrolysate of Green Peas Bromelain (PHGPB) has been successfully produced using a simple process [2] that aims to provide a nutritional solution with essential amino acids suitable to meet the protein needs of CKD patients. Our previous study shows that PHGPB offers an effective therapy for renal fibrosis by improving renal function parameters in the CKD rat model [3]. The delivery of PHGPB suppressed the expression of the SMAD 2, 3, and 4 genes but increased the SMAD 7 gene. PHGPB has promising antifibrotic effects on the TGF-1 level/SMAD2, SMAD3, SMAD4, and SMAD7 gene expression in glucose-induced MES13-SV40 cells. Furthermore, administration of PHGPB containing RGD peptide to renal mesangial lineage cells has been shown to reduce fibrosis parameters (fibronectin and TGF-beta), hence, PHGPB is expected to prevent renal fibrosis from occurring in CKD. The effective oral dose of PHGPB in rats is 200 mg/kg body weight/day, which is considered a reasonably high dose when converted to humans. It requires a large dose as it is primarily broken down in the digestive tract. However, oral administration of a drug can have a therapeutic effect on the target organ. In order to increase the bioavailability of PHGPB, it is necessary to develop a formulation that allows PHGPB to accumulate specifically at diseased sites. Many researchers have introduced nanoparticle-based delivery platforms that enables drugs or nutrition to reach their target organs effectively [4,5,6]. 

Tissue engineering is an advanced area of reparative medicine that emerged from the field of biomaterials development [7]. Basically, this technique repairs, improves and maintains the function of injured tissue or organ by combining cells, biologically active molecules, and scaffolds. The goal of tissue engineering is to bring together functional constructs that provide biological support to damaged tissue/organ for its proper restoration and regeneration [8,9]. Chitosan is a chitin-derived biopolymer that has shown great potential for tissue regeneration and controlled drug delivery [7,8,9,10]. Chitosan is the second most abundant natural polysaccharide biomaterial. Chemically it consists of -(1 4)-2-amino-2-deoxy-D-glucose and is a product of partially or fully deacetylated chitin. It is a natural biopolymer derived from crustacean shells such as crabs, shrimp, and lobster [11,12]. It is soluble in water and many organic solvents. It offers multiple locations and opportunities for chemical modifications. Most of the chitosan’s distinctive properties are attributed to the presence of a primary amine group found along its main chain. Chitosan can be modified by grafting pendant NH_2_ or OH functional groups without modifications in the polymer chain. Consequently, this natural biomaterial exhibits unique biocompatibility, biodegradability, and affinity for biomolecules, and has become popular for biomedical applications [13,14,15]. In addition to biocompatibility and biodegradability, non-toxicity without the formation of acidic degradation products is a broad-spectrum characteristic of chitosan-based biomaterials in biomedical applications [16]. We have successfully synthesized chitosan nanoparticles using the spray drying method [17] and applied them as a drug delivery system for alfa mangostine [18]. Chitosan is an efficient, inexpensive, and environmentally friendly source of nanocarriers among polymeric nanoparticles. The cationic properties, electrostatic properties, and biodegradability of chitosan NPs have drawn attention to its application as a facile oral and intravenous delivery of carrier drugs into host cells [19]. Researchers are optimistic about the use of chitosan NPs as part of a targeted drug delivery system for chronic kidney disease [19,20].

Therefore, the current study aims to introduce a chitosan nano delivery system for PHGPB to enable the controlled release of active ingredients. An effective delivery system is expected to improve the efficacy of renal fibrosis therapy in CKD, previously demonstrated by improvements in renal function parameters. The chitosan-PHGPB nano delivery system was prepared by the colloid spray-drying method. In addition, entrapment efficiency, drug loading, and water solubility were evaluated. 

## 2. Materials and Methods

### 2.1. Materials

The Green Peas Bromelain (Pisum sativum) powder was purchased from United State, Colorado (Trinidad Benham Corp. Denver, CO 80237, Split 200116, sieve MESH 120). Bromelain is an enzyme extract derived from the stems of pineapples. The chitosan and sodium tripolyphosphate (TPP) were received from Sigma Aldrich, Saint Louis, MO 63103, USA. A food grade MiliQ Water obtained from Arium Pro Ultrapure Water System, Sartorius Lab Instruments GmbH & Co. KG, Goettingen, Germany, and glacial acetic acid purchased from Merck KGaA, Darmstadt, German with CAS: 64-19-7.

### 2.2. Methods

**Figure 1:**
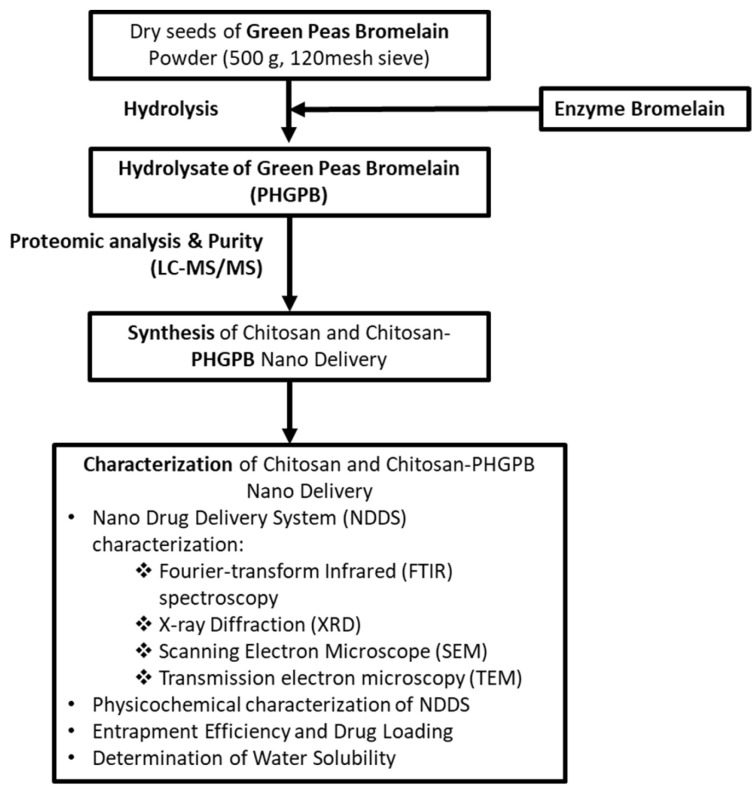
The scheme of experimental procedure.

The scheme of the experimental procedure of the present work is shown in Figure 1. There are two main steps of the experiments: (1) hydrolysis and characterization of Protein Hydrolysate of Green Peas Bromelain (PHGPB) and (2) synthesis and characterization of chitosan and chitosan/PHGPB nano delivery.

#### 2.2.1. Preparation of Protein Hydrolysate of Green Peas Bromelain (PHGPB)

The Green Peas Bromelain powder was hydrolyzed using the bromelain enzyme by simple method to obtain Hydrolysate of Green Peas Bromelain (PHGPB). Split green peas 20016 (The USA Trinidad Benham Corp. Denver, CO. 80237) were hydrolyzed using the previous method [21]. Dry seeds of green peas (500 g) were mashed and sieved through a 120 mesh sieve, then dissolved in 2000 mL of water. To the solution was added 10% (*w*/*v*) of bromelain [22] and then left at room temperature (25–30 °C) on a stirrer for 72 h. The solution was then centrifuged for 10 min at 2500 g at 4 °C (Refrigerated Centrifuge Tomy Portable MX-201). The supernatant was filtered using filter paper. 

#### 2.2.2. Protein Hydrolysate of Green Peas Bromelain (PHGPB) Characterization 

The molecular weight of the PHGPB was characterized using SDS-PAGE at a gel concentration of 15% and voltage of 90 V for 120 min. The proteomic analysis of PHGPB was conducted to investigate the peptide of LERGDT using LC-MS/MS. The amount of protein of PHGPB was calculated using the Bradford method [22]. The most reliable protein estimation is performed using a reference or a protein standard that has properties similar to the protein being estimated. Using bovine serum albumin (BSA) as the reference protein, the Bradford protein assays showed significant protein-to-protein variation; hence, the calculated result is an estimation of protein concentration. After obtaining the protein hydrolysate solution, the total amount of solution product, pH and protease was calculated based on the BSA equation. The amount of protein was calculated by dividing the absorbance of the hydrolysate sample at 50× diluted (0.02 sample + 0.98 aquadest) at wavelength A280 with the BSA equation.

#### 2.2.3. Synthesis of Chitosan and Chitosan/PHGPB

Chitosan polymeric particles were synthesized by the ionic gelation method using a sodium tripolyphosphate (TPP) as cross-linking agent. The TPP/Chitosan ratio was 1:5 (*v*/*v*) and was mixed in a magnetic stirrer at a speed of 1000 rpm for 12 h. There were two scenarios of chitosan concentrations, prepared at 0.1% and 0.25%, for a preliminary study on the spray drying process. The detailed operation of the colloidal spray drying method is explained elsewhere [4]. 

**Figure 2:**
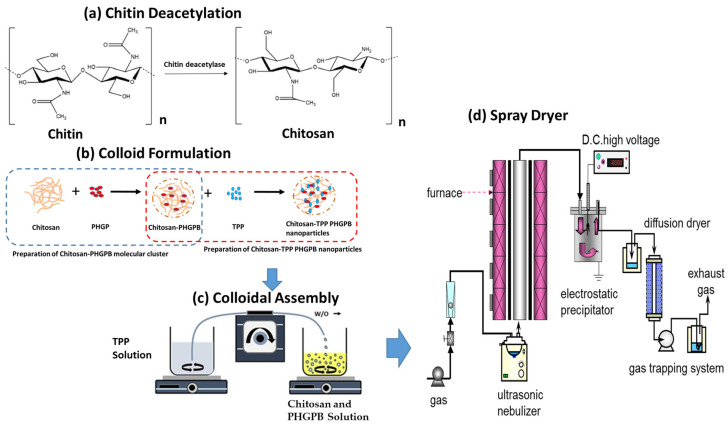
Formulation and Synthesis of chitosan-PHGPB Delivery System: (**a**) The chitin deacetylation, (**b**) colloid formulation of Chitosan-TPP PHGPB, (**c**) colloidal assembly, and (**d**) spray dryer apparatus.

The delivery system was synthesized by aerosol spray drying with the ultrasonic nebulizer and processed at a reactor temperature of 80 °C, and the DC high voltage powder collector at 100 °C using argon as the gas carrier (Figure 2). The bare chitosan was synthesized at concentrations of 0.1 wt.% and 0.25 wt.%, while the chitosan-PHGPB particles were synthesized to obtain a similar concentration of chitosan and PHGPB, 0.1 wt.% (Chitosan:PHGPB ratio of 1:1). Various synthesis process at different flow rate was formulated as presented in Table 1. First, the chitosan and PHGPB solution was mixed in the magnetic stirrer for 1 h at a speed of 1000 rpm. Then, the TPP was dissolved in a separate glass using a magnetic stirrer for 1 h at a rate of 1000 rpm. Furthermore, a micro pump transferred the mixture of chitosan and PHGPB into the TPP solution dropwise. The final solution was placed into the ultrasonic atomizer of the spray pyrolysis. The formation of particles depends on various process variables such as temperature, flow rate, and precursor solution concentration. In this study, the chitosan-PHGPB was synthesized with a spray pyrolysis process varied at different aerosol flow rates of 1, 3, and 5 L/m (Table 1). 

#### 2.2.4. Nano Drug Delivery System (NDDS) Characterization 

The chemical structure of the raw materials and their corresponding chitosan particles and chitosan-PHGPB particles were analyzed using Fourier-transform infrared (FTIR, Nicolet Is5 Thermo Scientific). The crystallinity of the as-prepared particles was observed using X-ray Diffraction (XRD, Bruker D8 Advance with Cu Kα with λ = 1.54060 Å). The morphology of particles was observed using Scanning Electron Microscopy (SEM JEOL). 

#### 2.2.5. Physiochemical of NDDS Characterization 

The chitosan and chitosan-PHGPB were characterized in size, size distribution, and suspension stability. To evaluate particle size, zeta potential, and polydispersity index (PI), freeze dried NPs were reconstituted in distilled water and measured using SZ 100 Horiba, Japan. The size of NPs was determined by a Particle Size Analyzer based on dynamic light scattering technique. The PI which is a dimensionless number indicating the width of the size distribution, was also measured. Zeta potential, an indicator of surface charge, which determines particle stability in dispersion, was also measured using the principle of electrophoretic mobility in an electric field. 

#### 2.2.6. Entrapment Efficiency and Drug Loading

Chitosan-PHGPB of 3 mg was dissolved into 15 mL MiliQ water, stirred for 4 h, and centrifuged at 2000 rpm for 20 min. The free PHGPB was dispersed into the supernatant. The supernatant was collected to measure the concentration of the free PHGPB drug using a UV-Vis spectrometer at the wavelength of 287 nm (maximum absorption wavelength for the PHGPB in water). The entrapment efficiency (EE%) and drug loading (%) were calculated using Equations (1) and (2):

(1) $$\mathrm{Entrapment}\;\mathrm{Efficiency}\;\left(\mathrm{EE}\;\%\right)=\frac{W_{t}}{W_{i}}\;x\;100\%$$

where W_t_ is the total amount of drug in the nano delivery suspension and W_i_ is the total weight of PHGPB drug added initially during preparation, and

(2) $$\mathrm{Drug}\;\mathrm{Loading}\;\left(\%\right)=\frac{W_{t}}{W_{\mathrm{nd}}}\;x\;100\%$$

where W_nd_ is the weight of nano delivery chitosan-PHGPB particle.

#### 2.2.7. Determination of Water Solubility

The water solubility of the drug delivery system was determined by dissolving chitosan-PHGPB and an equivalent amount of free drug in distilled water. The mixtures were stirred for 4 h and centrifuged at 2000 rpm for 20 min. The absorbance of the supernatant was measured by UV spectroscopy.

## 3. Results and Discussions

**Figure 3:**
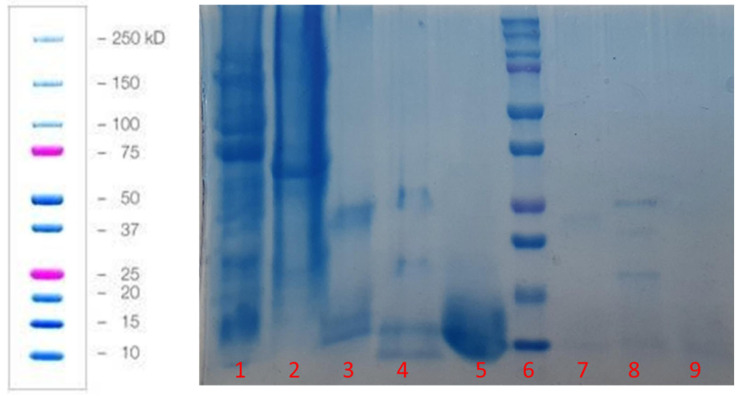
SDS PAGE of samples: 1. Pea powder, 2. Soybean powder, 3. Bromelain enzyme, 4. Pea hydrolysate solution, 5. Soybean hydrolysate solution, 6. Ladder, 7. Bromelain enzyme dilution 10×, 8. Pea hydrolysate solution 10× dilution, and 9. Soybean hydrolysate solution 10× dilution.

**Figure 4:**
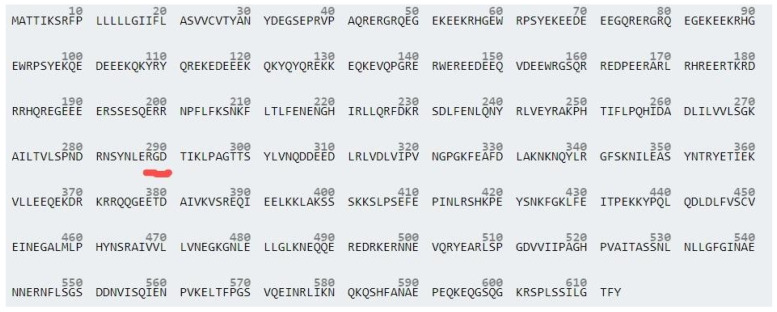
The position of RGD sequence in Pisum sativum Fraction Convicillin Q9M3X6 as highlighted in red line.

The protein of the obtained green peas powder was analyzed using the Bradford method. The calculated protein purity in PHGPB was 48.778 mg/mL, or approximately, 48%. The molecular weight of the hydrolyzed PHGPB observed using SDS-PAGE as shown in Figure 3. The proteomic analysis of PHGPB using LC-MS/MS to investigate the presence of peptide LERGDT is presented in Table 2 and the position of the RGD sequence in Pisum sativum Fraction Convicillin Q9M3X6 is presented in Figure 4. The results of the LC MS/MS analysis showed that one of the peas contains no. 10 Convicillin, Q9M3X6, Pisum sativum, with a molecular weight of 72 kDa. In the Convicillin protein, the RGD tripeptide sequence was obtained, which is in the LERGDT sequence, an active site in PHGPB in its effect as an antifibrosis in chronic kidney disease [23,24].

**Figure 5:**
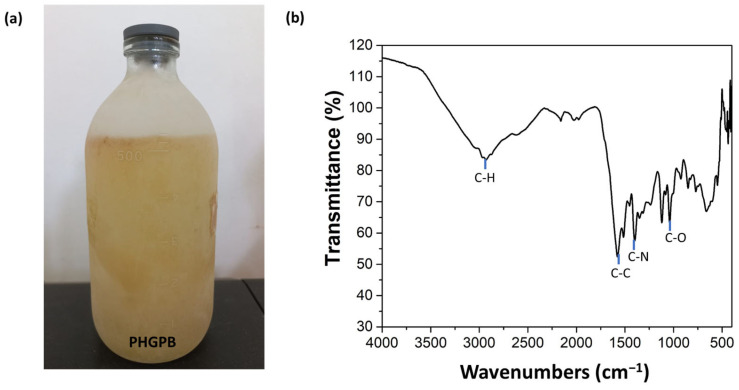
(**a**) PHGPB solution (**b**) FTIR of PHGPB.

Figure 5a shows the hydrolyzed PHGPB and its corresponding FTIR (Figure 5b). FTIR indicated that the bonding of C-H, C-C, C-N, and C-O represents the chemical structure of proteomic PHGPB.

**Figure 6:**
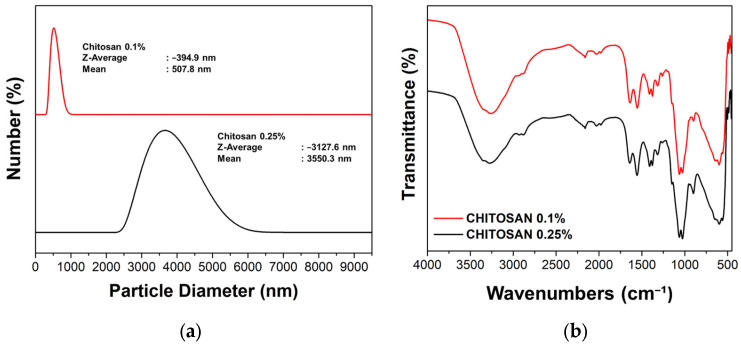
The particle size distribution (**a**) and FTIR (**b**) of chitosan 0.1 wt.% and 0.25 wt.%.

The powder obtained from scenario with chitosan concentrations of 0.1 wt.% and 0.25 wt.% were analyzed for their size distribution, as shown in Figure 6a and the corresponding FTIR (Figure 6b). This result showed that the synthesis of chitosan with a concentration of 0.1% by weight has a smaller size distribution compared to 0.25% by weight. This is a well-known phenomenon in synthesis made by a colloidal-spray dried method of smaller size at lower concentrations of precursor. Therefore, a further investigation of the synthesis of the chitosan-PHGPB at a concentration of 0.1 wt.% chitosan and a similar concentration of 0.1% by weight PHGPB at different aerosol flow rates was carried out.

**Figure 7:**
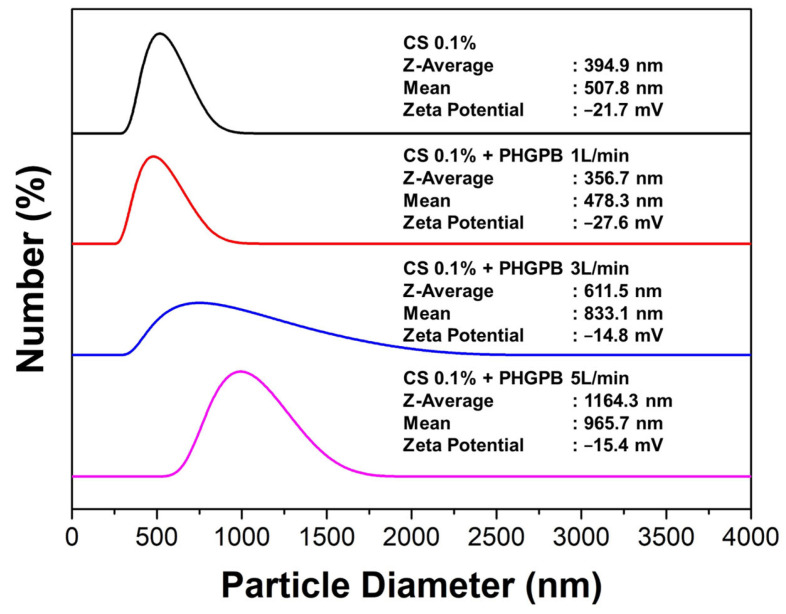
The size distribution and zeta potential of chitosan and chitosan-PHGPB process at a different flow rate.

The size of NDDSs plays a critical role in regulating their properties, such as conjugation power with drugs, crystallinity, and overall charge. The size of chitosan-PHGPB particles was compared to that of chitosan alone, as shown in Figure 7. The narrow size distribution was obtained from the aerosol flow rate of 1 L/min, with an average size of 356.7 nm. This result indicated that synthesis at a lower flow rate gave the aerosol a longer residence time in the reactor, consequently leading to a narrower particle size distribution. On the other hand, the peak of the size distribution of chitosan-PHGPB with flow rates of 3 and 5 L/m was broad, indicating that the particles were agglomerated in the suspension, observed from its lower zeta potential (−14.8 and −15.4 mV), compared to chitosan and chitosan-PHGPB with flow rate 1 L/m (−21.7 and −27.6 mV).

The surface charge of delivery nanoparticles determines their electrostatic interactions with plasma proteins, extracellular matrix components, and cell surfaces. The influence of the surface charge on plasma kinetics and biodistribution has been investigated by many researchers [25,26,27,28]. Arvizo RR et al. [25] found that particles with a neutral, negligible zeta potential and zwitterionic around −2.0 mV have a greater area under the curve (AUC) and slower elimination than do charged particles of the same diameter. Particles with positively charged particles, = +24.4 mV, have the fastest clearance [26,27,28]. However, positively charged formulations generally have the lowest plasma half-lives and AUCs, even when compared to free drugs. This can possibly be explained by high accumulation in the lungs, and by rapid aggregation with anionic blood species, resulting in emboli, thus, suggesting a preference for negatively charged particles in NDDS design [27,28].

In contrast, for tumor delivery purposes, meta-analysis shows a research trend demonstrating positively charged NPs have greater uptake than neutral ones, which in turn, perform better than negatively charged particles [27]. For long circulation and good cell affinity, nanoparticles require specific, sometimes mutually conflicting, surface charge requirements; this poses a dilemma for the development of polymer nanoparticles for controlled drug delivery [28]. In order to solve this problem, Huang W.F. et al. suggested the development of nano drug delivery systems (NDDSs) with both pH-responsive and switchable surface charge properties. The dilemma can be avoided by tailoring the composite nanoparticles with a core-shell structure designed to be negatively charged initially and to switch to a positive charge later. The negative charges of particles can be switched to positive charges gradually as the erosion of biodegradable polymer shells and exposure of the positively charged chitosan core. Thus, the obtained negatively charged chitosan-PHGPB particles provide a promising system for controlled drug delivery [29,30,31].

**Figure 8:**
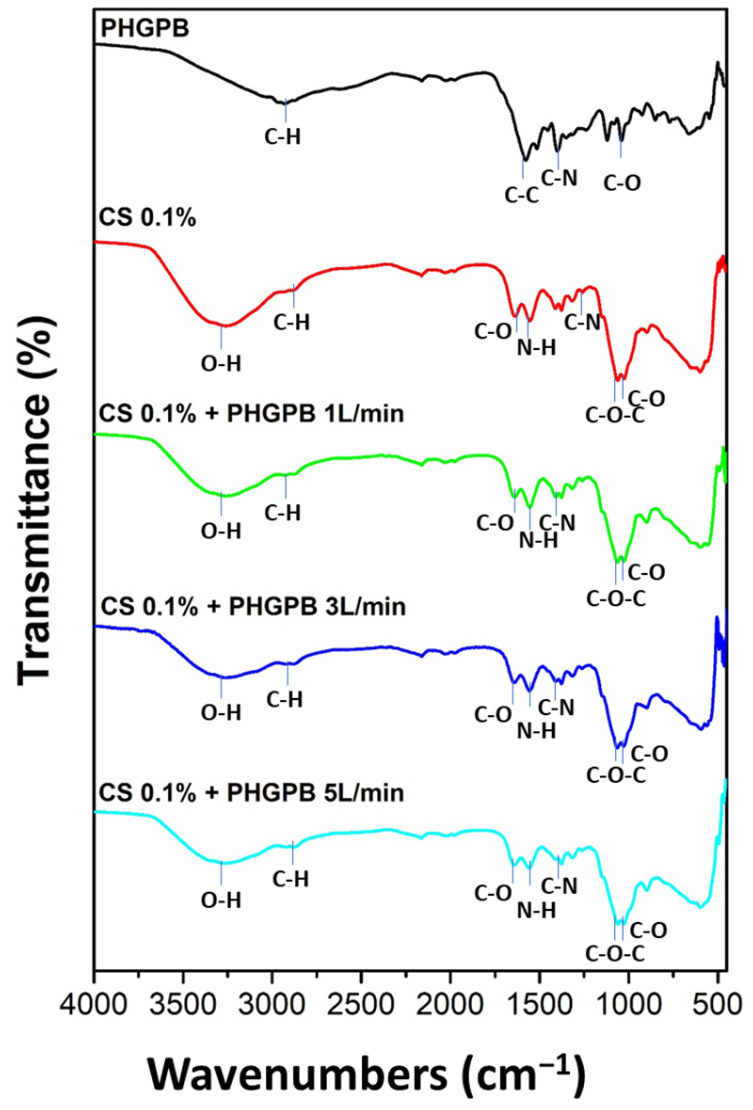
The FTIR of PHGPB, chitosan, and chitosan-PHGPB at various flowrate 1, 3, and 5 L/min.

Figure 8 shows the FTIR of PHGPB, chitosan, and chitosan-PHGPB at flowrates of 1, 3, and 5 L/min. This investigation aims to find whether the PHGPB had been entrapped in the chitosan during the spray pyrolysis process. The lower peaks of OH in the chitosan-PHGPB, compared to chitosan, indicated the presence of the PHGPB in the formulation.

**Figure 9:**
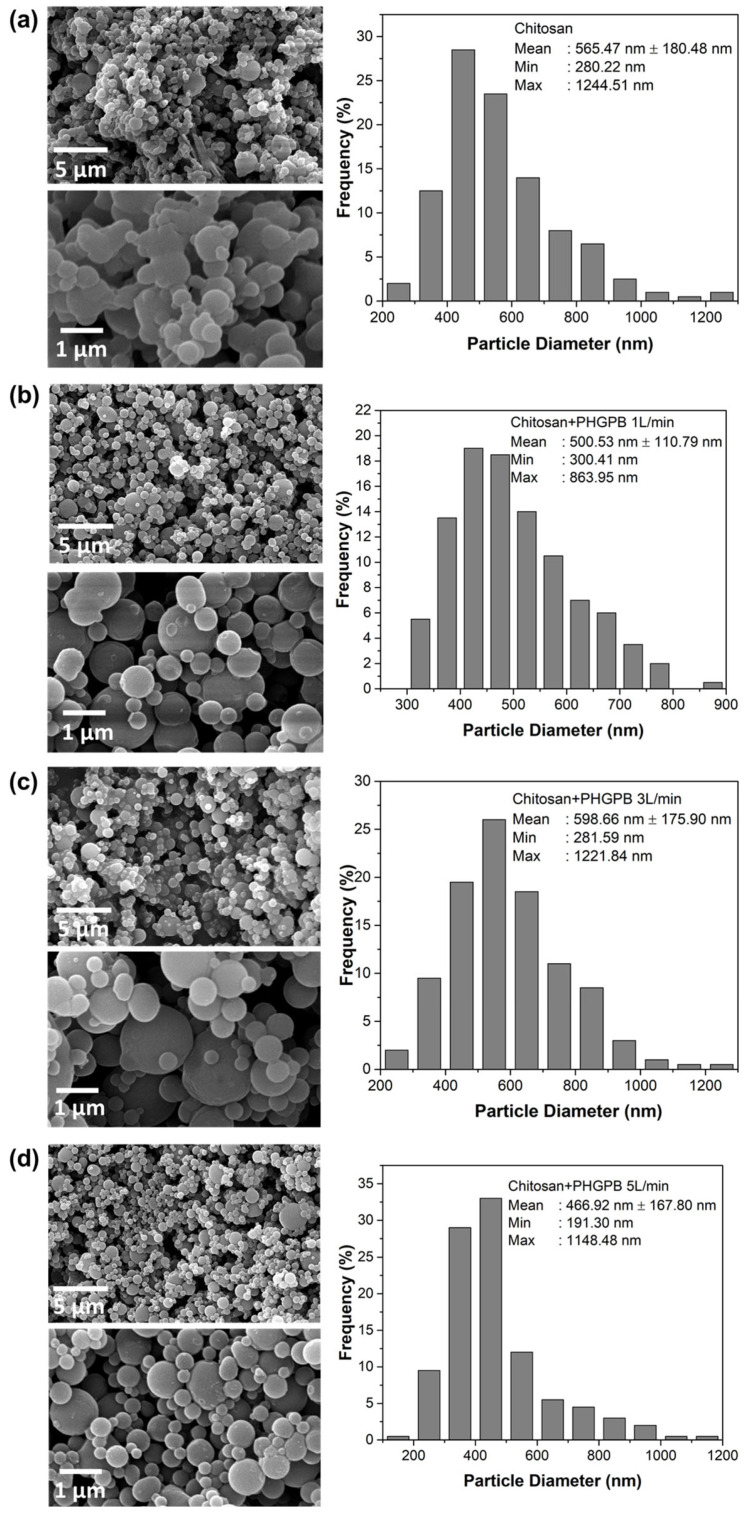
The SEM images and their corresponding size distribution of (**a**) Chitosan 0.1 % (**b**) Chitosan 0.1% + PHGPB 1 L/min (**c**) Chitosan 0.1% + PHGPB 3 L/min (**d**) Chitosan 0.1% + PHGPB 5 L/min.

**Figure 10:**
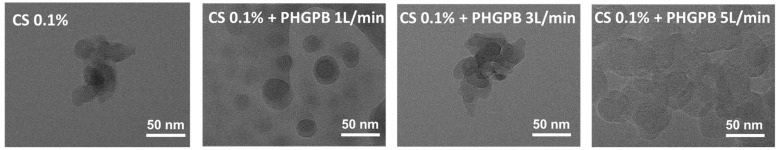
The TEM images of the Chitosan-PHGPB delivery system prepares at various aerosol flow rate in comparison to only chitosan.

Size distribution is the main determining factor of a NDDS’s effectiveness, as each organ’s capillary bed has a pore size exclusion limit. The most prominent feature of the SEM images in Figure 9 showed all polymeric nanoparticle particles were relatively spherical in morphology. The SEM images of chitosan showed aggregation of spherical particles during the spray drying process. However, particles of chitosan-PHGPB showed spherical morphology. A lower-sized distribution was obtained from the chitosan-PHGPB synthesized using a flow rate of 1 L/min with an average size of 500.53 nm. Similarly, the TEM images showed more a spherical morphology in the chitosan-PHGPB synthesized utilizing a flow rate of 1 L/min, which agrees with the SEM observations (Figure 10).

**Figure 11:**
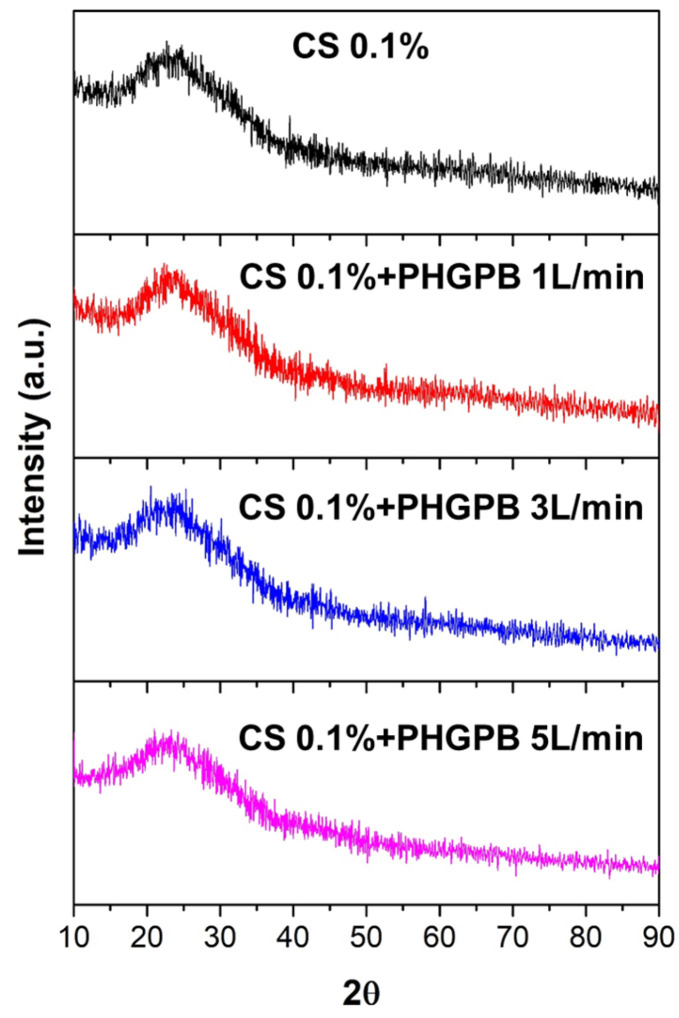
The XRD chitosan-PHGPB delivery system prepares at various aerosol flow rates in comparison with the chitosan.

The XRD spectra of the samples show that all chitosan-PHGPB and chitosan only particles remained amorphous (Figure 11). This result indicated that the additional PHGPB did not change the crystal structure of the particles.

**Figure 12:**
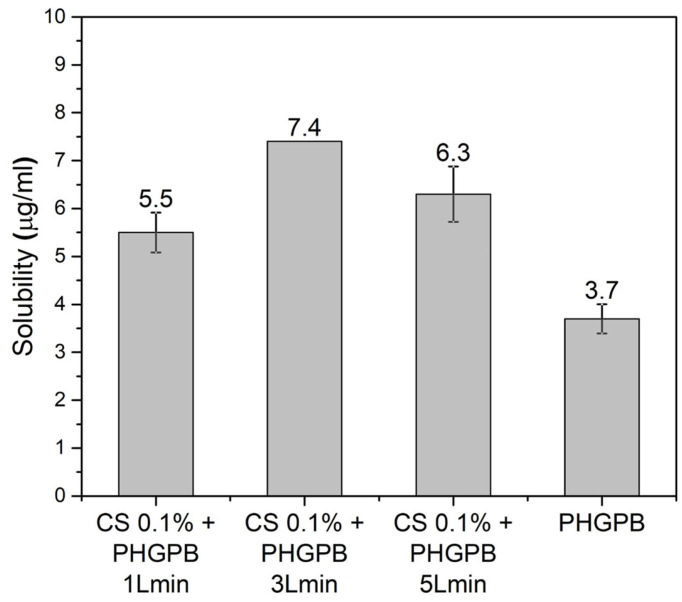
The solubility of the chitosan delivery system of PHGPB prepared at various aerosol flow rates in comparison with only PHGPB.

The PHGPB encapsulation efficiency (EE) and loading capacity (LC) of the particle delivery system at various processing aerosol flow rates 1, 3, and 5 L/min are shown in Table 3 and Figure 12. Cs 0.1% + PHGPB 1 L/min showed the highest EE (87%), but the lowest drug loading capacity. In contrast, the particles Cs 0.1% + PHGPB 3 L/min produced the lowest EE (83.84%), resulting in the highest loading capacity. Generally, the highest concentration of entrapped material or ingredients in each system affected the particle size. This was clearly observed for Cs 0.1% + PHGPB 3 L/min, the largest particle size compared to other samples [5].

## 4. Conclusions

In summary, chitosan-PHGPB particles prepared by colloidal-spray drying method were successfully formulated and evaluated. The homogeneous size and spherical morphology of NDs with an average size of 356.7 nm were obtained for the chitosan-PHGPB with a flow rate of 1 L/min. Similarly, the chitosan-PHGPB delivery system prepared with a flow rate of 1 L/min showed the highest entrapment efficiency (EE = 87%) with drug loading (4.61%). However, the highest solubility (7.4 µg/mL) obtained from CS 0.1% + PHGPB 3 L/min associated with the highest loading capacity (6.17%). The solubility of CS 0.1% + PHGPB 1 L/min and CS 0.1% + PHGPB 5 L/min were 5.5 and 6.3 µg/mL, respectively. It is suggested that the chitosan-based drug delivery system, applied to improve the bioavailability of poorly soluble PHGPB, which is limited by poor dissolution rates, can potentially provide biocompatibility, biodegradability, and non-toxicity properties. The chitosan-based drug delivery system also provides the most efficient and reliable synthesis method for extension of commercial production. Fundamental in vitro, ex vivo, in situ, and in vivo characterization methods used for most nanocarriers are needed. These should emphasize their advantages and limitations, as well as the safety, regulatory and manufacturing aspects that are possible limitations or challenges to transfer of nanocarriers from the laboratory to the clinic.

## Figures and Tables

**Table 1:** The samples code and description of the synthesis.

No.	Samples Code	Description
1	Chitosan 0.1%	Chitosan 0.1%
2	Chitosan 0.1% + PHGPB 1 L/min	Chitosan 0.1% + PHGPB, synthesis at flow rate 1 L/min
3	Chitosan 0.1% + PHGPB 3 L/min	Chitosan 0.1% + PHGPB, synthesis at flow rate 3 L/min
4	Chitosan 0.1% + PHGPB 5 L/min	Chitosan 0.1% + PHGPB synthesis at flow rate 5 L/min

**Table 2:** The protein Fraction of PHGPB in Pisum sativum observed using LC MS/MS.

No	Accession	Description	Coverage	Peptide	PSMs	Unique	Protein	AAs	MW (kda)	Calc.pl
1	Q5ZGL2	Seed albumin2 (Fragment) OS = Pisum sativum OX = 3888 GN = pa2 PE = 4SV = 1	67	3	6	1	1	51	5.7	8.5
2	P08688	Albumin-2 OS = Pisum sativum OX = 3888 PE = 2SV = 1	38	7	18	5	1	231	26.2	5.35
3	ADA161AT60	Non-specific lipid = transfer protein 1 OS = Pisum sativum OX = 3888 PE = 1 SV = 1	19	2	2	2	1	120	12.2	8.65
4	Q53WT6	P139 protein OS = Pisum sativum OX = 3888 GN = p139 PE = 2 SV = 1	19	1	1	1	1	74	8.2	6.48
5	Q76KV8	Short chain alcohol dehydrogenase A (Fragment) OS = Pisum sativum OX = 3888 GN = sadA PE = 2 SV = 1	10	2	2	2	1	277	29.2	6
6	PO2867	Lectin OS = Pisum sativum OX 3888 GN = LECA PE = 1 SV = 1	10	2	2	2	1	275	30.3	5.03
7	PO2855	Provicilin (Fragment) OS = Pisum sativum OX 3888 GN = LECA PE = 3 SV = 1	7	1	1	1	1	275	31.5	5.76
8	O24294	Legumin (Minor small) OS = Pisum sativum OX 3888 GN = LegS PE = 2 SV = 1	4	2	2	2	1	566	64.8	5.55
9	P15838	Legumin A2 OS = Pisum sativum OX 3888 GN = LEGA 2 PE = 3 SV = 1	2	1	2	1	1	520	59.2	6.62
10	Q9M3X6	Convicilin OS = Pisum sativum OX 3888 GN = cvc PE = 2 SV = 1	2	1	1	1	1	6123	72	5.6
11	P09918	Seed linoleate 95-Lipoxygenase-3 OS = Pisum sativum OX 3888 GN = LOX1.3 PE = 2 SV = 1	1	1	1	1	1	861	97.6	6.51

Note: PSMs: Peptide Spectrum Matches; AAs: Amino Acids; MW: Molecular Weight; Calc.pl: Calculated Protein Load.

**Table 3:** The Mean Entrapment Efficiency and Drug Loading of the chitosan-PHGPB particle.

Formulae	Entrapment Efficiency (%)	Drug Loading (%)
CS 0.1% + PHGPB 1 L/min	87.92 ± 0.91	4.61 ± 0.35
CS 0.1% + PHGPB 3 L/min	83.84 ± 0.00	6.17 ± 0.00
CS 0.1% + PHGPB 5 L/min	86.17 ± 1.26	5.28 ± 0.48

## Data Availability

Not applicable.
